# Potential Application of Living Microorganisms in the Detoxification of Heavy Metals

**DOI:** 10.3390/foods11131905

**Published:** 2022-06-27

**Authors:** Runqiu Chen, Huaijun Tu, Tingtao Chen

**Affiliations:** 1 Departments of Geriatrics, the Second Affiliated Hospital of Nanchang University, Nanchang 330031, China; chenrunqiu588558@163.com (R.C.); ndefy10061@ncu.edu.cn (H.T.); 2 Queen Mary School, Nanchang University, Nanchang 330031, China; 3 National Engineering Research Center for Bioengineering Drugs and Technologies, Institute of Translational Medicine, Nanchang University, Nanchang 330031, China

**Keywords:** heavy metals, dysbiosis, probiotics, molecular techniques, engineered bacteria, human health

## Abstract

Heavy metal (HM) exposure remains a global occupational and environmental problem that creates a hazard to general health. Even low-level exposure to toxic metals contributes to the pathogenesis of various metabolic and immunological diseases, whereas, in this process, the gut microbiota serves as a major target and mediator of HM bioavailability and toxicity. Specifically, a picture is emerging from recent investigations identifying specific probiotic species to counteract the noxious effect of HM within the intestinal tract via a series of HM-resistant mechanisms. More encouragingly, aided by genetic engineering techniques, novel HM-bioremediation strategies using recombinant microorganisms have been fruitful and may provide access to promising biological medicines for HM poisoning. In this review, we summarized the pivotal mutualistic relationship between HM exposure and the gut microbiota, the probiotic-based protective strategies against HM-induced gut dysbiosis, with reference to recent advancements in developing engineered microorganisms for medically alleviating HM toxicity.

## 1. Introduction

Rapid industrialization and urbanization have dramatically increased human exposure to heavy metals (HMs) [1], especially in developing countries. In Asia, high concentrations of HMs have been found in surface soil, drinking water and groundwater in China, Bangladesh, Vietnam, Thailand, Nepal and India [2]. In China, the total mass of HMs introduced in various waste at enormous magnitude is approximately 0.9 million tons each year [3]. Furthermore, as reported, an area of 34,000 km^2^ with a population of 30 million was covered by HM-polluted groundwater in six districts of India [4]. Recent accumulating epidemiological evidence suggests that high-level HM exposure causes severe damage to various organs and systems, including the kidneys, liver, the central nervous system (CNS), the reproductive system, and the hematopoietic system [5]. More than 20 types of HMs have been identified, among which cadmium (Cd), lead (Pb), and inorganic arsenic (As) were considered the most hazardous elements [6]. Based on the duration, HM exposure can be mainly classified into acute (1–14 days), intermediate (15–354 days) and chronic (≥365 days). Actually, compared to acute HM poisoning commonly induced by skin contact, the inhalation of large amounts of HM vapors, or drug misuse within a short time, chronic HM poisoning resulting from inconspicuous daily exposure through food, water, air or skin is more commonly encountered in the clinic and poses a serious threat to public health [7]. At present, chelation therapy has been the mainstay treatment for HM poisoning and related disorders; however, the use of conventional chelating agents is associated with common side effects such as adverse drug reactions, gastrointestinal distress, loss of essential metals, and strong clinical nephrotoxicity [8,9]. For this this reason, the exploration of more specific and safer therapeutic strategies for HM intoxication is the need of the hour.

The human gut microbiota, as a “superorganism” composed of 400–1000 bacterial species, fulfills many crucial roles in maintaining human health including nutrient metabolism, maintenance of mucosal integrity, immune system modulation, and protection against pathogens [10,11]. Such dynamic bacterial communities are susceptible to changes in the host conditions induced by extrinsic substances; as a result, disturbance of the gut microbiome called dysbiosis can affect the health status of the host and may end up in disease conditions [12,13]. Recently, a strong bidirectional relationship between HM exposure and gut microbiota was proposed by studies from rodent models to other advanced models [14,15,16]. It is evident that HM exposure, especially chronic exposure alters the gut microbiome trajectory and phylogenetic diversity, which may thus perturb the metabolic and physiological functions of gut microbiota, consequently, leading to the rise of many pathological conditions and toxic symptoms after HM exposure [17,18]. Reciprocally, the gut microbiota act as the primary defense against HM toxicity by affecting the intestinal HM absorption and metabolism, whilst enhancing the fecal HM excretion [19,20]. In the meantime, probiotics have received special attention due to their remarkable HM-binding capability. Generally, probiotics are termed as mono or mixed cultures of selective viable microorganisms that confer health-promoting benefits to the host by improving the balance of the intestinal micro flora [21], which traditionally include those microorganisms derived from the species of Bifidobacterium, Lactobacillus, Streptococcus, Enterococcus, Clostridium, Bacillus, and *Escherichia coli* (*E. coli*) [22]. Some classic probiotic strains, especially Lactobacillus, Bacillus, Bifidobacterium and Clostridium species have been shown to adapt strong HM resistance through alteration of the physiological conditions [23,24], or expression of HM-binding peptides/proteins [25,26] or detoxification enzymes involved in HM biotransformation [27] to reverse HM-induced dysbiosis, thereby delivering a defense against HM intoxication. However, since the health benefits of these strains appeared to be limited and ineffective, next-generation probiotics, especially bioengineered bacteria, have emerged as novel protective and therapeutic bioagents in many fields [28,29]. Great progress has been made in the application of engineered probiotics to deliver therapeutic molecules to target tissues, or express specific enzymes for local cleavage of prodrugs, or function in tandem with the host immune system to induce tolerance [30]. To date, recombinant bacteria have been used in attempts for the treatment of metabolic disorders such as diabetes [31], phenylketonuria (PKU) [32], autoimmune diseases such as inflammatory bowel disease [33], and cancer [34]. Based on this, the aid of engineered bacterial transformation technology opens up possibilities to design and develop genetically modified microbes with the desired characteristics and functionalities towards HM detoxification, which represent a promising generation to alleviate chronic HM toxicity and fill the gap in current therapeutic strategies.

In this review, we highlight the health risks of HM bioaccumulation in the human body and the underlying mechanisms of their action. We also summarize the bidirectional relationship between HM exposure and the gut microbiota, as well as the potential use of probiotics for the remediation of HM toxicity. In particular, this review outlines the current uses and possible future trends of novel-engineered bacteria for HM detoxification, which may help to provide an intriguing new therapeutic approach for chronic HM poisoning and related toxic symptoms.

## 2. Heavy Metal Poisoning: Health Risks and Mechanisms of Toxicity

HMs are inorganic elements with a high density of more than 5 g/cm^3^ [35]. Based on their toxicity, HMs can be classified into essential and non-essential groups. The essential HMs including components such as iron (Fe), zinc (Zn), copper (Cu), and calcium (Ca) are cofactors in various biological processes, but their excessive accumulation in the human body also leads to harmful effects. While the non-essential HMs such as arsenic (As), mercury (Hg), cadmium (Cd), and cobalt (Cr) have no known benefits for the human physiology, they are lethal to living animals even at low concentrations [36]. These highly toxic HMs persist in various industrial, agriculture, domestic and medical waste, leading to various degrees of HM pollution in water, soil, and air that cause considerable HM accumulation in animals and plants. Unconsciously, along the cycles of the food chain, humans are in turn exposed to HMs by consuming contaminated plants and animals and this long-term exposure has been known to pose a serious threat to public health [37]. For this reason, dietary intake has been recognized as the most common route for HM exposure. This is exemplified in the observations that the blood level of Hg was dramatically high in large populations of seafood consumers, especially those living around metallic mineral deposits with diets composed of local freshwater fish, and those dependent on marine mammals as their main food source in Arctic regions [38,39]. According to the WHO ranking of the top ten toxic pollutants, As, Cd, lead (Pd), and Hg are ranked as the most hazardous chemicals of global health concern [40]. Therefore, this section provides a comprehensive illustration of the human biotoxic effects of As, Cd, Pd, and Hg and the underlying mechanisms of their toxicity.

As is a semimetal that is mainly exposed to humans via the intake of contaminated water with arsenical pesticides [41]. A large number of epidemiological studies have reported that in China, Thailand, West Bengal, Bangladesh, Inner Mongolia, Hungary, Mexico, Chile, Argentina, and Finland, various pathogenic conditions were observed in large populations that were under high-level As exposure in their drinking water, including neurologic and neurobehavioral disorders, hematologic disorders, cardiovascular and peripheral vascular disease and developmental anomalies [42,43]. This highlights the fact that As exposure is indeed associated with increased health risks. Acute As toxicity is associated with hyperesthesia in extremities, abdominal patellar reflexes, abdominal cramping, and abdominal electrocardiograms [44]. When there is chronic exposure, it is clinically manifested as prominent skin lesions of pigmentation and keratosis with high specificity in diagnosis [45]. Moreover, exposure to inorganic As in the forms of arsenite (As III) and arsenate (AsV) is a major risk factor for cancers of the liver, lungs, skin, and bladder [46]. In fact, the carcinogenic role of As (III) results from the inactivation of essential enzymes. Hydrogen is replaced by As (III) from the thiol group to form a dihydrolipoyl-arsenite chelate complex, which blocks the activity of pyruvate oxidase and α-ketoglutarate dehydrogenase enzymes, ultimately disrupting ATP production and causing cell damage [47]. Inorganic As further leads to significant inhibition of protein kinase B (PKB), a well-known enzyme responsible for various cellular metabolic pathways, thus contributing to multiple clinical symptoms related to As intoxication [48].

Tobacco smoking and the consumption of staple foods and seafood are the primary exposure sources of Cd [49]. Regardless of the route of exposure, once accumulated in the human body, Cd is considered non-biodegradable owing to its long biological half-life of 17 to 30 years without excretion [50]. Multiple organs and systems are the vulnerable targets for Cd intoxication, but the lungs and kidneys are especially affected, associated with symptoms of breath shortness, pulmonary edema, and even respiratory failure in severe cases [51]. According to the International Agency for Research on Cancer of the USA, Cd has been listed as a #1 category human carcinogen [49]. The rise of reactive oxygen species (ROS) or free radicals in Cd-exposed cells is indicated to be the major mechanism of Cd toxicity [52]. The excess ROS causes damage to the mitochondrial membrane and interferes with the mitochondrial electron transfer chain, also induces a lipid peroxidation process that leads to protein dysfunction and loss of membrane integrity. Subsequently, these biochemical changes result in abnormal apoptotic events that stimulate cell proliferation within the tissues [53].

Pb is a ubiquitous pollutant that possesses wide applications such as in lead-based paints, cosmetics, gasoline, pipes, and battery casings [54]. Despite Pb exposure generally having declined since the late 1970s, Pb poisoning in children still remains a serious global concern nowadays. Studies conducted by National Health and Nutrition Examination Surveys (NHANES) have reported that excessive Pb levels (>10 mg/dL) have been found in large populations of children in the United States [55,56,57]. Importantly, children and infants are more susceptible to low-level Pb exposure than adults. Many published studies have documented the Pb-associated adverse effects in children with elevated Pb blood level—decreased IQ levels, retarded neurobehavioral development, speech and language handicaps, and various impaired cognitive development [58,59,60]. In specific, Pb is prone to form an insoluble phosphate that deposits in the skeletal bones of approximately ninety-five percent [61]. For adults, acute Pb toxicity is represented by abdominal pain, headache, renal dysfunction, reproductive defects and hematological disorders [62]. The prime mechanism of Pb-induced hematological toxicity is the inhibition of the enzymes involved in heme production such as porphobilinogen synthase and ferrochelatase due to Pb accumulation in erythrocytes thus resulting in microcytic anemia [63]. Moreover, Pb is known to produce ROS in many systems that oxidize low-density lipoprotein (LDL) and induce endothelial inflammation, subsequently leading to an increased risk of thrombosis and atherosclerosis in Pb-exposed individuals [64].

Hg is highly bio-accumulative in water resources, usually in the organic form of methylmercury (MeHg) that causes severe disturbance to aquatic lives. Thus, the ingestion of contaminated fish and crustaceans is one of the major routes for human Hg exposure due to the bio magnifications through the food chain [65]. Even in developing countries, it is estimated that up to 10–15 million miners are still suffering from chronic Hg intoxication [66]. Once taken up into the human body and reaching the bloodstream, the ethyl and methyl groups present confer MeHg high hydrophobicity and lipid solubility to ensure easy passage through the blood-brain barrier (BBB) and gain entry into the brain. As a result, MeHg intoxication is responsible for CNS symptoms including irritability, tremors, memory problems, and even dysfunction of vision or hearing [67]. If absorbed into the placenta, MeHg will impair embryonic development that leads to disorders such as autism. Apart from dietary ingestion, the inhalation of Hg vapors is also extremely dangerous as it could accumulate in the lungs and cause pulmonary damage, nausea, diarrhea, vomiting, skin rashes, hypertension, or tachycardia [68]. At the subcellular level, the biochemical basis for Hg toxicity is the depletion of glutathione (GSH), one of the most crucial anti-oxidative enzymes that protect the cells from oxidative stress and inflammation. Therefore, Hg exposure is associated with the overproduction of free radicals and elevated lipid peroxidation, which causes the mitochondrial damage and cellular dysfunction that give rise to multiple clinical manifestations [69].

## 3. The Conventional Therapeutics for Heavy Metal Poisoning and Their Limitations

The liver is the main organ responsible for HM detoxification in the human body. There are two pathways involved in the detoxification process, known as the Phase I and Phase II liver-detoxification pathways. As the fist line of defense against HM toxicity, the Phase I pathway consists of a catalogue of enzymes referred to as the cytochrome P450 family that convert toxic xenobiotics into less toxic forms via the reactions of oxidation, reduction and hydrolysis. In Phase II, also called the conjugation pathway, water-soluble side groups (e.g., cysteine, glycine or a sulfur molecule) are added to the toxic chemicals to enhance their hydrophilicity for subsequent excretion via watery fluids such as bile or urine [70]. However, when excessive HMs enter the human body in a short period of time that overload the liver detoxification pathways, toxicity will be built up and cause acute poisoning. In the clinic, chelation therapy is the primary treatment for this condition. The basis for chelation therapy is a process in which small organic molecules typically bind to metal ions by forming coordination complexes involving interactions with oxygen, sulfur or nitrogen atoms [71]. According to the US National Library of Medicine, five chelating agents including Dimercaprol (British Anti-Lewisite, BAL), 3-Dimercapto-Propanesulphonate (DMPS), Sodium-calcium EDTA (CaNa2-EDTA), Dimercaptosuccinic acid (DMSA), and Penicillamine are most prescribed for the treatment of HM intoxication [72]. However, disappointingly, some common adverse effects have been reported during the course of chelation therapy. Headache, fever, gastrointestinal distress, muscle pain, high/low blood pressure, and even worsening conditions of respiratory and heart failure, permanent kidney damage, convulsions or seizures have been reported in chelator-treated patients [73,74]. Although the management of acute HM poisoning is a medical emergency, its occurrence is indeed rare in clinical practice except in cases of suicide. In fact, as HMs have wide domestic, industrial, technological, agricultural, and medical applications, its chronic exposure through various anthropogenic activities, particularly occupational exposure is more common today as a threat to public health. Due to its non-negligible adverse effects, chelation therapy is not the best-fitted choice for the treatment of chronic HM poisoning. To date, no effective therapeutic agents with fewer side effects have been found or developed to prevent further HM absorption into the system, or inactivate the HM bioavailability during chronic exposure. Therefore, there is an urgent need to explore effective methods to remedy the current defects in conventional therapy for the treatment of chronic HM intoxication.

## 4. The Gut Microbiota: A Vital Mediator in the Heavy Metal-Induced Toxicity

The crucial role of gut microbiota in maintaining human health has been precisely investigated by numerous studies. As a pivotal regulator of the digestive system, the gut microbiota participates in the metabolism and storage of nutrients, xenobiotics and drugs; it also assists in antimicrobial protection, immunomodulation, and even maintains the structure and integrity of the gastrointestinal tract [75]. There is clear evidence that many prevalent metabolic and immune diseases originate from the intestine as a result of the disruption of normal gut microbiota, named dysbiosis [76,77,78]. The GI tract is the main site by which HMs enter the human body. Recently, there is growing consensus that HM exposure may also contribute to dysbiosis, highlighting a pivotal mutualistic relationship between HM exposure and the intestinal microecology [79]. Therefore, in this part, we aimed to discuss the putative impacts of HMs on gut microbiota at the functional level and how this disturbance increases the subsequent health risks.

Previous researchers already noted that HM exposure had direct effects on gut microbiota composition, generally leading to altered bacterial diversity, a loss of specific health-promoting bacteria, or an increase in the pathogenic microbiome. In most studies, it was reported that the abundance of Proteobacteria and Firmicutes decreased while Bacteroidetes increased at the phylum level after HM exposure [18,79]. Concerning the suborder level, it was demonstrated that acute MeHg exposure resulted in alterations of generic diversity of the orders Desulfovibrionales, Peptococcaceae, and Helicobacter [80]. Further, the perturbation of the gut microbiome was also observed after Pb exposure: an over-abundance of those within the families Desulfovibrionaceae, Barnesiella, and Clostridium XIVb, whereas a sharp decrease in the relative proportion of the orders Lactococcus, Enterorhabdus, and Caulobacterales in Pb-exposed individuals [81]. Cd, Pb, Cu, and aluminum (Al) treatments in mice reduced the relative abundance of the species *Akkermansia muciniphila* (AKK) in a metal-specific and time-dependent manner [82].

As a result of the complete ablation of the gut microbiota composition, HM exposure interfered with the metabolic profiles of the gut microbiota at the functional level, invariably leading to changes in the microbial metabolic products associated with energy harvesting, inflammatory response, and the generation of oxidative stress (Table 1) [83]. This is because various metabolic products, including secondary bile acids, short-chain fatty acids (SCFAs), vitamins and other cofactors, as well as several harmful metabolites such as cresol and indole can be produced by intestinal flora through the digestion and biotransformation of amino acids [83], polysaccharides [84] and primary bile acids [85]. Thus, HM-induced minor compositional changes in the gut microbiota can be linked to major alterations in the concentrations of these bacterial metabolites. For instance, in accordance with the previous finding that *Bifidobacterium* is a vibrant mediator for vitamin biosynthesis, Chi et al. found that the over-representation of *Bifidobacterium* after As exposure resulted in an elevated expression of vitamin biosynthesis genes, which was hypothesized to be a resistant mechanism of the gut microbiota to counteract As-induced toxicity [86]. A recent report has also shown that the concentrations of numerous metabolic substances including fatty acids, bile acids, amino acid derivatives, indole-containing compounds, glucuronide, isoflavone, and carnitine conjugates were remarkably changed following As exposure, which was closely linked to the structural and compositional changes of the colonic microbiome [87]. Similar results have also been found in MeHg exposure, that is, MeHg-induced perturbation of the gut microbiome subsequently caused the decrease in the concentrations of palmitic, oleic, and stearic acids, meanwhile increasing the concentration of glycerol in exposed mice and fish [88].

There is clear evidence that HM-induced compositional and metabolic disruption of the gut microbiota leads to a series of downstream effects that contribute to various diseases, such as obesity, allergies, diabetes, autism, Crohn’s disease, and inflammatory bowel disease [96]. A conceivable explanation for this has been proposed that the gut microbiota and their metabolites are highly involved in multiple metabolic pathways and the physiological processes of different organs and systems. An elegant example proved that the short-chain fatty acids (SCFAs) produced by gut microbiota serve as the ligands of the G-protein-coupled receptor (GPCR), the inhibitors of histone deacetylases, and the energy substrates for colonocytes and gluconeogenesis [97,98]. Based on this, it can be hypothesized that HM-induced perturbation of SCFA production exerts an influence on energy metabolism, cancer genesis, and even nervous system function. Notably, the gut microbiota and its metabolites also function in tandem with the host immune system in an elaborate manner [99]. Consequently, immune responses and even some immunological diseases can be triggered by an HM-induced disruption of the balance between the host immune system and gut microbiota [100].

In particular, the gut–microbiome–brain axis is of great significance in the connection of small changes in microbial communities with severe neurobehavioral disorders. Under pathological conditions, some metabolites and virulence factors of the gut microbiota such as spermidine, D-lactic acid, and lipopolysaccharide (LPS) can penetrate the mucosal blood vessels through “gut leakiness”. Subsequently, these bacterial metabolites and virulence factors are prone to pass across the BBB thus inducing encephalopathic effects and triggering neurobehavioral responses [101]. Moreover, the penetrated bacteria and their metabolic products also restrain or mimic the synthesis of neurotransmitters and hormones, such as serotonin, norepinephrine (NE), gamma-amino butyrate (GABA) and dopamine, which then intervene with the normal host neuroendocrine responses [102]. As a consequence, an HM-induced disturbance of the gut microbial structure and metabolic profiles will aggravate some neurobehavioral disorders, including cognitive dysfunction, nerve injury, and autism spectrum disorders (ASD) [103,104].

## 5. Probiotic-Based Protective Strategies against Heavy Metal-Induced Gut Dysbiosis

**Figure 1:**
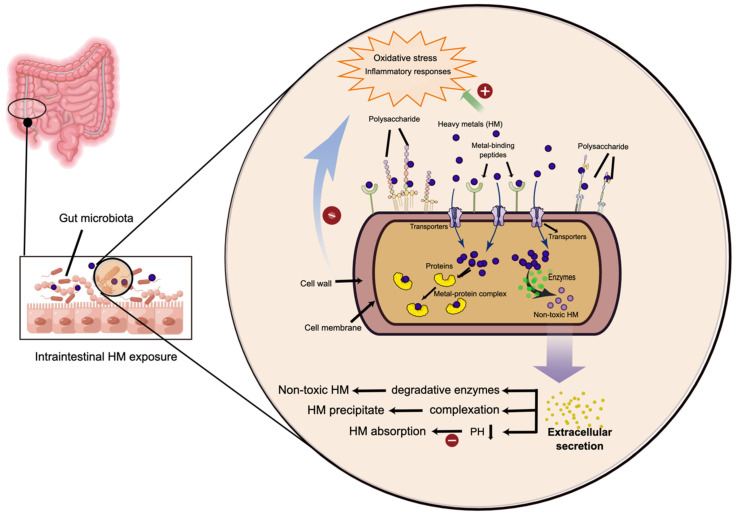
The underlying mechanisms of heavy metal (HM) detoxification by probiotics. (1) Cell wall binding sites for HMs: bacterial cell walls contain different polysaccharides and HM-binding proteins that serve as potential binding or biosorption sites for metal ions. (2) Intracellular sequestration of HMs: metal ions enter the cytoplasm by active transportation or passive diffusion via transporters, then bind with the intracellular metal-chelating proteins to form protein–metal complex or transformed into nontoxic forms undergoing enzymatic reactions. (3) Extracellular secretion for HM detoxification: various substances or molecules can be secreted by probiotics for three purposes: convert HM into less toxic/adsorbable forms; form complex-precipitation with HMs; decrease intestinal PH and subsequently inhibit the HM absorption into epithelial cells. (4) Counteraction of the HM-induced oxidative stress and inflammatory responses. By Figdraw (www.figdraw.com accessed on 26 March 2022).

It is now well established from a variety of studies that the relationship between gut microbiota and HM exposure is mutual and bidirectional. HM exposure alters the microbiome composition and metabolomic profiles of the gut microbiota, conversely, the gut microbiota has strong HM-metabolizing capabilities to limit HM absorption while increasing HM excretion from the human body (Table 2). For this reason, the application of probiotics is considered as a promising approach to alleviating HM-induced toxicity, which raises great prospects to be the next-generation therapeutics for HM intoxication. From the existing literature, several mechanisms have been explained so far as modes of probiotic resistance against HM intoxication, with the following properties of particular importance: (1) they directly affected the absorption and metabolism of HMs within the intestine; (2) they have intense abilities to bioaccumulate, bind, or transform HMs via various enzymatic reactions; (3) they are considered as strong antioxidants with immune regulatory capability; (4) they can reverse HM-induced disruption in the relative abundance of gut microbiota (Figure 1). Based on the four resistant strategies, this section outlines the current evidence showing the extent to which the probiotics exert protective effects against HM toxicity.

Within the human intestinal tract, there are large populations of bacteria performing a decisive role in HM absorption and metabolism. In most cases, transforming toxic metals into biologically inaccessible forms is a prime mechanism underlying the HM-metabolizing capabilities of probiotics. This is exemplified in the findings that some potential probiotic strains *Xanthomonadaceae*, *Comamonadaceae*, *Pirellula*, *Cloacibacterium*, and *Deltaproteobacteria* FAC87 convert methylated Hg to the less soluble form Hg^0^, which reduces its absorption in the gastrointestinal tract [95,105]. Likewise, *Lactobacillus plantarum* (*L. plantarum*) TW1-1 has been found to convert Cd into a less absorbable form that is poorly incorporated in the alimentary canal [23]. During the process, the methylation of HMs is a pivotal step to metabolize HMs into lower solubility, which was proved in the cases of sulfate-reducing bacteria (SRB), Fe-reducing bacteria, *methanogens*, and *Desulfovibrio* spp. [79]. Furthermore, it is an intriguing finding that the treatment of *L. plantarum* CCFM8610 and CCFM8661 in mice increased the production and excretion of bile acids, meanwhile enhancing the elimination of HMs from the mice intestine [23,24]. In addition, *L. plantarum* LC-705 and *Propionibacterium freudenreichii* have been shown to inhibit the absorption of Pb and Cd by decreasing the intestinal PH [106].

It has long been recognized that gut microbiota has potent capabilities to bioaccumulate or bind HMs via metal-specific binding proteins, or transform HMs into nontoxic forms via various enzymatic reactions, thus facilitating HM excretion from the host system and reducing the overall HM accumulation [25]. On the one hand, various metal-chelating agents produced by gut microbiota play a crucial role in the binding and sequestration of target HMs. For instance, siderophores, an iron (Fe) ion-chelating peptide synthesized by *Pseudomonas* [25] have been identified to chelate the ions of Pb, Cd, Hg, Cr, and As all through the formation of insoluble complexes with these HMs [107]. Other classic examples of metal-chelators yielded by probiotics include the hydrogen sulfide from SRB [108] and the oxalate from *Oxalobacter formigens (O. formigens)* that are capable of forming precipitates with toxic metal ions [109]. By forming these HM-protein complexes that deposit in the intestine, toxic metals could thus be isolated from absorption into the epithelial cells. On the other hand, diverse enzymatic transformations have been recognized as crucial resistant approaches for probiotics to combat HM harmfulness in recent studies. *Faecalibacterium prausnitzii (F. prausnitzii)* is a commercialized probiotic that is renewed by its capability to synthesize an As-detoxifying enzyme called methyltransferase [27]. Similarly, it is reported that *Bacteroides* and *Faecalibacterium* secrete a reductase ArsC converting toxic As (V) into less-toxic As (III) within the intestine [110]. Another gene coding an As oxidase enzyme (aioB) has been found in the gut microbiota of alcoholic cirrhosis patients [111].

As a vital modulator of the immune systems, gut microbiota has a strong antioxidative capability to tolerate the acidic conditions in gastrointestinal fluid and an immune regulatory capability to repress pathogen development [112] of note, the HM-triggered overproduction of the oxidative stress and downstream inflammatory responses have been considered as the most important mechanisms responsible for HM-induced toxicity [113,114]. Therefore, the antioxidative and immune regulatory effects of probiotics are crucial to counteract the noxious consequence under HM exposure. This can be clearly seen in the cases of *L. plantarum* CCFM639 and *Bacillus Coagulans (B. coagulans)* that in-vivo administration of these two strains could attenuate HM-induced generation of toxic hydroxyl radicals and ROS, mainly by promoting the expression of antioxidant-related genes to synthesis antioxidative enzymes [115]. In the aspect of immune modulation, *L. plantarum* CCFM639 [116], CCFM8610 [23], and *L. brevis* 23017 [117] have been reported to relieve the deleterious effects of HMs by reducing the levels of proinflammatory cytokines, thus alleviating the HM-induced intestinal inflammatory responses. Moreover, *Nocardia* and *Bacteroidales* could also produce specific immune regulatory substances with antimicrobial activities against MeHg exposure [118].

In addition, accumulating evidence has shown that probiotic intervention could reverse the compositional changes of gut microbiota resulting from HM exposure, thereby re-establishing the structural balance and integral diversity of gut microbiota to deliver defense against HM toxicity. An example of this is the study carried out by Zhai et al. in which the inoculation of probiotic strain *L. plantarum* CCFM8610 reloaded the *Flavobacterium* and *Pseudomonas* content of gut microbiome in HM-treated fish [23]. Likewise, Probiotic *Pediococcus pentosaceus* GS4 and *Akkermansia muciniphila (A. muciniphila)* increased the richness of the gut microbiota especially the *Lactobacillus* and *Clostridiales* content in Cd-exposed mice [19,119]. Encouragingly, a clinical trial has confirmed that oral supplementation of *Lactobacillus rhamnosus (L. rhamnosus)* GR-1 in yogurt decreased the blood concentration of HMs both in pregnant women and children [120], mainly through restoring the gut microbiome composition after HM exposure that was termed as “gut remediation”.

## 6. Potential Application of Engineered Bacteria in the Detoxification of Heavy Metals

Although the majority of natural probiotics do not display infectivity or pathogenicity, the safety concerning the application of probiotic-based bioagents still remains a grey area. Contrary to expectations, some probiotic strains, particularly the enterococci were reported to pedal a string of genes related to transmissible antibiotic resistance. *Bacillus cereus* was also identified with the potential to produce emetic toxins and enterotoxins [121]. Moreover, it was observed that probiotic intervention caused some common side effects of deleterious metabolic activities, systemic infections, gene transfer, and excessive immune stimulation in susceptible individuals [122]. In fact, the HM-resistant properties are limited to particular microbial species, and it is necessary to combine several different strains together through oral consumption to warrant the therapeutic efficacy. It is therefore likely that complicated relationships and conflicts still exist in applying different probiotic strains, which arouse public concerns over the health risks associated with probiotic-based therapy [123].

In virtue of the serious defects in natural-origin probiotics, engineered probiotic microbes with the ideal characteristics and functionalities have emerged as an intense focus in recent years. Some strains such as *Lactic acid bacteria (LAB)* and *E. coli Nissle 1917 (EcN)*, have limited therapeutic effects themselves in practice, whereas, the introduction of synthetic biology engineering is helpful to strengthen the overall effectiveness of these probiotics [124]. In detail, the survival and byproduct formation of LABs can be apparently limited in the presence of bacteriophages in the gut microbial ecosystem, for instance, the virulent Lactobacillus phage Lb338-1 was reported to exert a negative influence on the beneficial effects of the Lactobacillus strains [125]. To overwhelm this defect, specific phage-resistant bacteria have been developed by genetic modification, and further studies have shown that the mutant-engineered strain not only preserved the same probiotic features as the original parent strains, but was also equipped with antimicrobial properties against virulent pathogens [126]. On the other hand, EcN, one of the best commercially used probiotic strains in many European countries [127], has been particularly documented for the treatment of patients with ulcerative colitis (UC) [128]. The main mechanism of its action is thought to be its capability to induce the production of human β-defensin 2 (HBD2) in intestinal epithelial cells [129]. However, EcN appeared to be ineffective in the remission of Crohn’s disease (CD), due to the primary lack of defensin synthesis in the intestinal tract of CD patients [130]. Based on this, an engineered EcN strain with defensin-producing and secreting functions has been constructed, which was capable of synthesizing human α-defensin 5 (HD5) and HBD2 derivatives that might act as a delivery vehicle for these defensin-deficient patients [131].

To date, aided by the continuous development of synthetic biology and recombinant DNA technology, the therapeutic implications of genetically engineered microorganisms (GEMs) have made remarkable strides. Although it is still in the trial phase, a range of metabolic disorders, autoimmune and inflammatory diseases, infectious diseases, cognitive dysfunctions, and cancer have been targeted by the delivery of engineered probiotics and have achieved satisfactory effects of amelioration [29,132,133,134]. In principle, engineered microorganisms exert their therapeutic effects mainly through the delivery of a vaccine or drug, modulating the host immune response, imitating surface receptors or aiming at particular toxins or pathogens within the intestine to perform in situ activities [135]. Compared to traditional pharmacotherapy, engineered probiotic mediated treatments own many irreplaceable advantages such as high stability, increased standby time, reduced systemic exposure, lower delivery cost and local targeting to mucosal surfaces.

In the past decades, the application of GEMs for HM removal has attracted considerable attention due to their low cost, flexible adaptability, and eco-friendly properties. GEMs with intense degradative capacity have been widely used for the bioremediation of HMs in groundwater, soil, and activated sludge conditions. Generally, the construction of potent HM-resistant GEMs is mainly based on two strategies: (1) surface functional complexes with a high HM-binding capacity for biosorption, (2) metal ions are transported into the cytoplasm and subsequently processed by storage systems for enhanced intracellular bioaccumulation [136]. In this part, emphasis will be placed on various HM-chelating peptides/proteins, transport and storage systems and related molecular technologies that are commonly employed for the surface-adsorption and bioaccumulation of HMs.

### 6.1. Surface-Displayed Proteins/Peptides for Heavy Metal Biosorption

**Figure 2:**
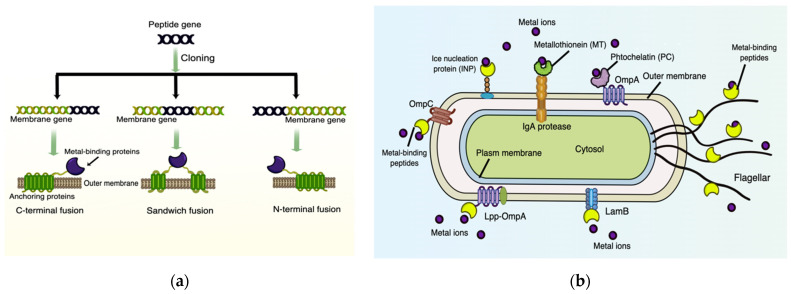
The procedure and principle of surface engineering towards metal adsorption in Gram-negative bacteria. (**a**) The coding DNA of target metal-binding peptides/proteins can be obtained from genome or plasmid DNA. After cloning it is transformed into the genome of host bacteria via fusion with the coding gene for an anchor protein (membrane protein) by one of the three different recombinant ways. Subsequently, by specific induction, the recombinant gene undergoes transcription, translation, and translocation into the cell surface. Based on different genetic recombinations, the target metal-binding peptide can be immobilized with the anchoring protein in the cell surface by C-terminal fusion, sandwich fusion or N-terminal fusion; (**b**) commonly used surface display systems in Gram-negative bacteria are described as follows: outer membrane proteins (OMPs): OmpA, OmpC, LamB, Ice nucleation protein (INP) and Lpp-OmpA; autotransporter: IgA protease; flagella. By these surface display systems, various metal-binding proteins/peptides can be anchored onto the outer membrane to adsorb specific metal ions. Metallothionein (MT) and phytochelatin (PC) are the most investigated metal-binding peptides that have been anchored to IgA protease [86] and OmpA [85], respectively. By Figdraw (www.figdraw.com accessed on 3 Arpil 2022).

Biosorption is a metabolism-independent process by which metal ions can be adsorbed and immobilized onto the cell surface via physicochemical reactions or surface precipitation [83]. Recently, the application of molecular biology has enabled the display of foreign metal-binding proteins/peptides or other tailor-made binding proteins on the cell surface to exhibit enhanced HM biosorption. The basic principle of different bacterial display systems is fusing the heterogenous HM-binding proteins (target protein) with naturally occurring anchoring proteins (carrier protein) via three connection approaches, C-terminal fusion, N-terminal fusion, or sandwich fusion (Figure 2a), but meanwhile maintaining the independent spatial structure and biological activity of both proteins [137].

Notably, the surface display systems are entirely different in Gram-negative and Gram-positive bacteria owing to their distinct cell structures. Gram-negative bacteria have an extra outer membrane thus making membrane-spanning anchoring proteins necessary as a functional carrier for connection with the target external proteins. The most frequently used surface-display systems in Gram-negative bacteria contain outer-membrane proteins (OMPs), autotransporters, and the surface organelles fimbriae/flagella [137]. Among the various OMPs, ice nucleation protein (INP), Lpp-OmpA, OmpA, OmpC, LamB have been extensively applied to design engineered Gram-negative bacteria with HM adsorption capability [138]. In the autotransporter system, IgA protease β-domain is the best-known protein that has been successfully used to immobilize mouse metallothionein I (MT) protein on the cell surface [139]. Furthermore, the FimH protein, an integral part of type 1 fimbriae has been explored to display HM-binding peptides for the bioadsorption of Cd^2+^ and Ni^2+^ [140]. (Figure 2b). Whereas for Gram-positive bacteria, on account of their thick peptidoglycan layer and lack of outer membrane, the surface proteins (target proteins) are covalently linked to the peptidoglycan cell wall instead of membrane-spanning. Staphylococcal protein A (SpA) is the most-investigated anchor protein in Gram-positive bacteria [141]. Several HM-binding proteins/peptides, including two polyhistidyl peptides (HHHEHHH and HHHHHH) and screened variants of cellulose-binding domain (CBD) have been surface displayed on Gram-positive bacteria via the SpA system for HM removal [142].

In recent years, an increasing number of novel metal-binding peptides/proteins have been identified and expressed on bacterial surfaces via various display systems for HM removal. Metallothioneins (MTs) and phytochelatins (PCs) are the most classic HM-chelating proteins with desirable affinity and specificity towards Pb, Hg, As, Cd, and Ag in most genetic engineering cases [143,144]. PCs have been surface-displayed on *Moraxella* spp., *Pseudomonas putida (P. putida)*, *Caulobacter crescentus* and *Mesorhizobium huakuii* that exhibited desirable biosorption for Hg and Cd [145]. Surface engineering of MTs on *Ralstonia eutropha* and *E. coli* cells indicated a 15–20 times higher Cd accumulation compared to the wild-type strains [144,146]. Apart from MTs and PCs, a surprising array of novel metal-binding domains have been explored for their potential application in HM removal. For instance, three copper-binding peptides NRWHHLE, NAKHHPR, and SPHHGGW [147], two Cd-binding peptides polyhistidine His12 [148] and SynHMB [149], three Pb-binding proteins PbrR, PbrR691, and PbrD [150] were surface-expressed in different strains to adsorb HMs from the contaminated sites.

### 6.2. Transport and Storage Systems for Heavy Metal Bioaccumulation

**Figure 3:**
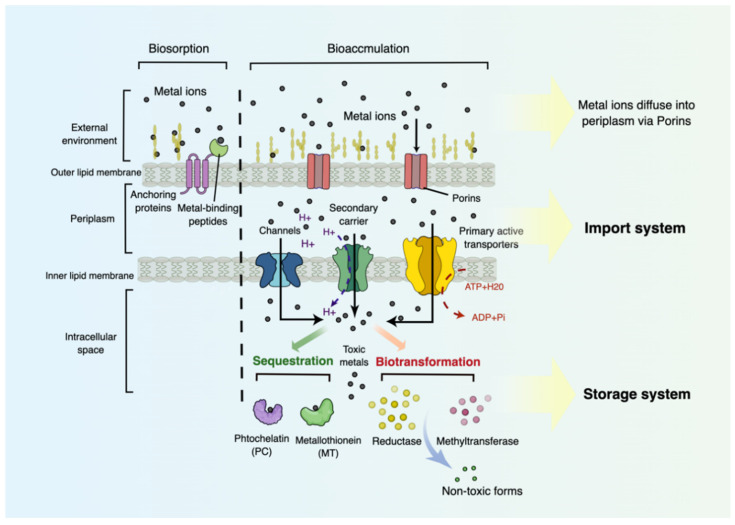
Import system and storage system adapted in Gram-negative bacteria towards bioaccumulation. Biosorption is indicated in the left side. The import system used in bioaccumulation includes primary active transporters (requiring NTPs such as ATP), secondary carriers (requiring a proton concentration gradient), and channels (no energy needed), by which metal ions are imported across the inner lipid membrane into the cytoplasm and undergo the process of the storage system. The storage system is responsible for HM sequestration by attachment to different metal-binding proteins/peptides (represented by PCs and MTs) or HM biotransformation by various detoxifying enzymes (represented by Hg reductase and As methyltransferase). By Figdraw (www.figdraw.com accessed on 1 April 2022).

Bioaccumulation is another probiotic-adapted mechanism to tolerate HM stress. It is a metabolically active process responsible for transporting HMs into intracellular space and subsequently secluding them by metal-chelating proteins/peptides or biotransforming them via enzymatic reactions. The most well-known import systems for HMs include three major transporter classes: primary active transporters, channels, and secondary carriers [151] (Figure 3). In detail, primary active transporters spontaneously carry HMs into cells against a concentration gradient by hydrolysis of ATP/GTP. Secondary carriers are capable of translocating cationic HMs into cytoplasm driven by the proton-motive force (PMF). Channels are energy-independent transporters that engage in the passive diffusion of HMs into the cells along their concentration gradient [152]. In recent years, vast experimental attempts have explored the utilization of several ion import systems to strengthen HM uptake by genetic engineering. Primary active transporters MntA and cdtB from *L. plantarum* and TcHMA3 from the flowering plant *Thlaspi caerulescens* have been recombinantly expressed in engineered strains for the efficient import of Cd [153,154]. Secondary carriers NixA and its homologs have been used to improve Ni and Co uptake, and Hxt7, Pho84 from *Saccharomyces cerevisiae* have been identified for As removal [155,156]. Moreover, researchers have discovered channels of homotetramer glycerol facilitators (GlpF) [157] and homolog Fps1 [158] with a specific affinity toward As, and MerT/P, MerC, MerE, and MerF [159] for the transport of Hg in the bioaccumulation process.

Immediately after the import of HMs into the intracellular space, multiple storage systems serve as the key to sequestrating or detoxifying HMs for the release of the HM-stimulated oxidative stress and inflammatory responses. In most cases, the HM-storage systems refer to genetically encoded metal-binding proteins/polymers (MBPs), or a catalogue of enzymes that biotransform metal ions into less toxic forms. In addition to the best-known metal-binding proteins PCs and MTs, Hpn (UniProt P0A0V6) from *Helicobacter Pylori (H. pylori)* [160], and (UniProt Q9FCE4) from *Streptomyces coelicolor* [161] have been identified to bind Ni in the process of bioaccumulation. On the other hand, A Hg resistance gene (mer) operon coding for a Hg reductase that converts Hg^2+^ into volatile Hg is an elegant example of enzyme-mediated HM storage systems. More tellingly, this Hg resistant system aids in the Hg^2+^ import followed by enzymatic reduction of toxic Hg^2+^ into non-toxic Hg^0^, and ultimately leads to Hg^0^ diffusion outwards from the cells [162]. Several recombinant strains, including *Acidithiobacillus ferrooxidans* with over-expressed merC gene, *E. coli* with transferred merT–merP genes, and *D. radiodurans* with cloned merA gene have shown an ideal capability to detoxify Hg^2+^ [163,164]. In addition, arsM gene encoding S-adenosylmethionine methyltransferase for the conversion of toxic inorganic As to less toxic volatile trimethylarsine (TMA) has been obtained from *Rhodopseudomonas palustris* [165]. Overexpression of the arsM gene in *Sphingomonas desiccabilis* and *Bacillus idriensis* have exhibited promising As detoxification [166].

### 6.3. In Vivo Attempts for Heavy Metal Detoxification by Engineered Strains

In attribution to the successful application of GEMs in the field of environmental HM bioremediation, biomedical workers were inspired to explore the feasibility of developing engineered bacteria as biological medicines for HM poisoning. In this aspect, surface display techniques and various HM transport/storage systems might be effective tools to design tailor-made GEMs with intense detoxification capabilities, which may be directly inoculated into the intestine by oral consumption, thereby counteracting the toxic effects of HMs within the intestine. However, up to now, only very few engineered bacteria have been developed or applied in vivo as a biological treatment against HM poisoning. After the present probe into pre-clinical research work, the following is a brief description of three currently available pieces of research on animal models that relate to this topic.

In 2018, Chan and his coworkers developed a novel whole-cell biosorbent for Pb by displaying PbrR, a well-investigated protein with Pb-binding capacity, on the cell surface of *E. coli* using Lpp-OmpA as the anchoring motif. In vitro, the results of atomic absorption spectroscopy (AAS) suggested that the PbrR-displayed cells had a four-times higher binding of Pb than non-PbrR cells, effective at both neutral and acidic pH. In vivo, a pre-experiment revealed that no noxious effects or pathogenic potential were observed after oral administration of PbrR-displayed cells on male Kunming (KM) mice. In order to determine the Pb-binding capacity of the PbrR-displayed *E. coli*, Pb^2+^ was supplied in the forms of lead acetate, saturated lead in PbrR-displayed *E. coli*, and unsaturated lead in PbrR-displayed *E. coli* to male KM mice at a dose of about 20 μg Pb/mouse daily for more than 15 days (human maximum Pb tolerable intake: 3 µg per day for children and 12.5 µg per day for adults). Encouragingly, the Pb concentration deposited in murine blood and bone was significantly reduced in all mouse groups treated with PbrR-displayed *E. coli*. compared to the Pb-only group. Furthermore, the metabolization of essential metals (Ca, Mg, Fe, Zn, and Cu) was not affected by the oral administration of PbrR-displayed *E. coli* [167].

In 2019, the team of Minrui Liu constructed two surface-displayed *E. coli* strains for selective adsorption of Hg^2+^ and its derivative MeHg, respectively [168,169]. For Hg^2+^ removal, a novel Hg^2+^-binding peptide encoded by the sequence of CysLysCysLysCysLysCys (CL) was surface-expressed on the *E. coli* BL21 by anchoring to the N-terminal region of the ice nucleation protein (INP-N). Similar to the last report, the in vitro AAS tests indicated a four-fold higher Hg^2+^ adsorption in CL-displayed *E. coli* than the control strain. Furthermore, the CL-displayed *E. coli* exhibited a strong specificity toward Hg^2+^ in the co-existence of other metal ions (Cd^2+^, Pb^2+^, Ni^2+^, Zn^2+^ and Cu^2+^). In vivo, *Carassius auratus (C. auratus)* were fed with engineered *E. coli* added to the fish food at a dose of 2 × 10^8^ CFU/g for 10 days (stage 1), and then were given 0.1 mg/kg Hg^2+^ exposure for 30 days (stage 2). It was shown that the Hg accumulation in fish muscles was reduced by 51.1% and the amount of Hg excretion in fish feces increased by 56.5% in the engineered *E. coli*-treated group compared with the non-fed group. Interestingly, it was observed that the Hg-induced increase in the relative amount of *Vibrio* spp. was reduced by 8.02% and the decrease in *Cetobacterium* was reversed by 12.00% after the oral administration of CL-displayed *E. coli* [168].

In the second study, engineered *E. coli* W-1 with surface-displayed CL peptide was constructed by the same method used in the last study. The results of the in vitro test showed that the CL-displayed *E. coli* W-1 exhibited a maximum MeHg adsorption rate of 96.3 ± 0.8%, whereas that of the original strain W-1 was less than 30%. Furthermore, the CL-displayed *E. coli* W-1 had a specific affinity towards MeHg when other metal ions (Cr^3+^, Zn^2+^, Cd^2+^, Ni^2+^, Cu^2+^, and Pb^2+^) persisted in the condition. The design of the in vivo experiment was similar to that of the study mentioned above, in brief, a 10-day inoculation of the recombinant *E. coli* at a level of 1 × 10^9^ CFU/g followed by a 30-day MeHg feed at a dose of 0.05 mg/kg on *C. auratus.* After DNA extraction from the intestinal contents of each individual fish, qPCR was used to detect the colonization and persistence of the engineered *E. coli* W-1 in fish intestines. The results indicated that, compared to the non-detectable proportion of engineered *E. coli* W-1 in the gut of the fishes from the control group, in the groups fed with engineered strains, the proportion of engineered E. coli W-1 accounted for 0.5% of the total bacterial community in the fish guts at the end of the in vivo experiment. This revealed that the engineered bacteria successfully colonized in the *C. auratus* intestine and persisted for an adequate period until the end of the 30-day MeHg feed, thus giving rise to the following result concerning MeHg reduction in fish tissues. As expected, compared to the untreated group, the accumulated MeHg concentrations in fish tissue were decreased by 36.3 ± 0.7% and the amount of MeHg excretion in feces was increased by 36.7 ± 0.8% in the groups treated with CL-displayed *E. coli* W-1 [169].

Taken together, the data reported here both support the idea that oral administration of surface-engineered bacteria is an effective approach to adsorb HMs in the intestine, thus reducing HM accumulation in the body and protecting animals from HM-induced toxicity. Collectively, the following findings should be highlighted from the present studies, which may provide new ideas for us to understand how GEMs aid in the intra-intestinal detoxification of HMs. Firstly, it should be noted that Lpp-OmpA and INP, the anchoring systems previously used for in vitro bioremediation, were also effective in immobilizing PbrR and CL proteins on the cell surface for in vivo application. Secondly, the evidence from these studies demonstrates that the recombinant bacteria could persist and vitally function for HM biosorption at different PHs, even in the extreme acidic conditions simulating the stomach. For instance, the amount of adsorbed Pb by PbrR-displayed *E. coli* was still 6.2-fold higher than undisplayed *E. coli* at pH 3.0 [167]. Thirdly, in all three studies, no noxious effects on animal growth nor intestinal epithelial invasion were observed after oral administration of engineered strains, which provides an experimental basis for safety assessment concerning their in vivo application. Further, the common property of the three engineered bacteria refers to a selective affinity towards the target HM even in the co-existence of other metals. This feature highlights one of their advantages, that is, the blood levels of physiologically essential metals will not be affected by their oral administration, overwhelming the conventional chelating agents with a side effect of essential metal loss. Last but not least, another noteworthy finding is that the oral administration of engineered *E. coli* W-1 reversed the HM-induced compositional alterations of gut microbiota [114]. A possible explanation for this might be that the host strain *E. coli* W-1, a commensal bacteria isolated from the fish intestine, has an innate probiotic property to counteract the HM toxicity by rebuilding the microecology. It can therefore be assumed that if probiotic species can be chosen for genetic engineering, their inherent beneficial properties will strengthen the detoxification effect of engineered bacteria and accelerate the process of intra-intestinal removal of HMs. In conclusion, the combination of these findings, at least preliminarily, suggests that the surface-display technologies conferred commensal bacteria- or probiotic-enhanced capability for in vivo HM adsorption, which lays the groundwork for future research into GEM-based preventive or therapeutic bioagents for HM intoxication. Finally, the most important source of weakness in the current studies needs to be illustrated here. The three in vivo experiments were not based on the model of chronic HM exposure, so fails to specify whether GEMs have the potential to be orally supplied for the treatment of chronic HM intoxication.

## 7. Current Limitations and Future Prospects of GEMs

Despite the utilization of GEMs for dietary consumption for the treatment of various diseases making great progress in food and medicine industries, there are still inevitable limiting factors hindering the large-scale application of GEMs. In particular, gene migration that weakens the functional stability of GEMs and relevant safety issues regarding human intake are of special concern. On the one hand, under stressful and competitive conditions, such as the internal environment within an animal body, the risk of gene migration including point mutations, fragmentary insertions or deletions, and even the loss of plasmids is likely to cause the loss-of-function effects on these engineered strains, which increases with the persistence period of its administration and colonization [170,171]. This is exemplified in a rat-model experiment where overall 40–65% of *Lactobacillu sparacasei (L. paracasei)* BL23 cells lost their engineered plasmids when passing through the intestine [172]. Even during in vitro growth, similar genetic loss was observed in engineered *Salmonella typhimurium (S. Typhimurium)* during a 60 h growth in a mouse tumor model [173]. Therefore, the development of retention systems that control the total and relative expression levels will be essential to restrict the spread of gene migration in GEMs. Tools such as a CRISPR–Cas9-based system that automatically restricts the expression of a synthetic circuit, have been generated most recently to improve the stability and therapeutic capacity of GEMs in vivo [174]. However, far greater control over loss-of-function gene mutations is likely to be necessary for any long-term GEM applications as diagnostics and therapeutics.

On the other hand, another major concern of GEMs and their clinical use is the safety issues. In spite of the well-known prohealth effects of engineered probiotics, some adverse risks have also been reported with respect to their use in humans. Generally, there are three common concerns in relation to the safety of GEMs: (1) the risk of disease occurrence in susceptible individuals, such as the cases of bacteremia reported in children with short gut syndrome after *Lactobacillus* GG intervention [175] and cases of endocarditis caused by *Lactobacillus rhamnosus (L. rhamnosus)* consumption [176]. (2) The potential metabolic or noxious effects on the gastrointestinal tract, for instance, the accumulation of conjugated bile acids that lead to malabsorption was observed in patients with short small bowel syndrome after the administration of *Lactobacillus acidophilus (L. acidophilus)* and *Bifidobacterium* species [177,178]. (3) The antibiotic-resistance transfer between probiotics and pathogenetic bacteria, including the encoded resistance to chloramphenicol, tetracycline and erythromycin, which have been found in the species of *L. plantarum*, *L. fermentum, L. reuteri* and *L. acidophilus* [179]. For this reason, screening of GEMs for their virulent traits and potential pathogenicity is essential to evaluate their safety prior to in vivo use. Moreover, it is necessary to develop molecular methods for the exclusion of antibiotic resistance. Furthermore, ideally, population-based surveillance for the occurrence of adverse effects should be conducted during any trial employing an engineered probiotic strain, especially in a list of patients with premature infants, immune compromise, short bowel syndrome, and central venous catheters [180]. In addition, nowadays the regulatory category for premarket approval of probiotic products is still incomplete, which might lead to unassured quality standards of the product contents when provided to the end-users [181]. Hence, it is quite imperative to establish a stringent safety policy to precisely address the issues of efficacy, safety, labels, and claims concerning GEM use.

As a matter of fact, engineered probiotics for therapeutic purposes, particularly those tentatively tested in clinic, have just reached the surface in regard to the whole potential and complexity of the use of genetic engineering. As it is different from in vitro growth in an ideally controlled medium, the usually oxygen- and nutrient-poor environment within the mammalian body is far more suboptimal and complex. The development of convenient and realistic in vitro tests is therefore the key to evaluating the in vivo potency of GEMs and predicting their clinical effectiveness. To date, advanced systems including organ-on-chip microfluidics [182], ex vivo organ growth techniques [183], and a simulator of the human intestinal microbial ecosystem (SHIME) fermenter system [184] have emerged that could possibly achieve better accuracy in estimating the in vivo efficacy of engineered bacteria. On the whole, GEMs have an underestimated and profound potential to contribute to clinical diagnostics and therapeutics in the future.

## 8. Conclusions and Perspectives

On the question of probiotic-based strategies, one thing needs to be explained clearly. Owing to the different mechanisms between acute and chronic HM exposure that result in distinct clinical symptoms, probiotic- or GEM-based strategies are not likely to be used as optimized therapeutics for acute HM poisoning and are only theoretically suitable for the prevention or treatment of chronic HM intoxication. As indicated previously, a pivotal mutualistic relationship between HM exposure, gut microbiota, and intestinal microecology has been suggested. HMs exert their noxious effects by altering the microbiome composition and metabolomic profiles of the gut microbiota, leading to a series of downstream responses that contribute to toxic symptoms in humans. By contrast, gut microbiota and some probiotic strains residing in the gut have developed strong abilities to alleviate HM toxicity through a series of resistance strategies, which indicates an intense potential to be the next promising generation for HM detoxification, especially for HM toxicity associated with chronic exposure. Whereas the action of natural microorganisms harbors multiple defects, GEMs start to gain significant attraction as a novel biomass for HM removal and bioremediation. The introduction of genetic manipulation strategies, especially the surface display techniques as well as various HM transport and storage systems enhancing the surface-adsorption and intracellular bioaccumulation of target HMs in GEMs, which opens up new approaches to create metal-specific biosorbents. Indeed, in vivo application of GEMs for HM removal is still a novel field, but encouragingly, several investigations in animal models have been the first attempts to thoroughly examine the oral consumption of GEMs on HM detoxification. Three engineered strains have been designed with different metal-binding peptides/proteins expressed on the cell surface, which exhibited desirable adsorption efficacy toward Pb, Hg, and MeHg in mice and fish models. Considering the status of global HM exposure, the further application of GEMs may help to provide feasible, low-cost preventive or therapeutic agents for chronic HM poisoning in humans.

Nevertheless, only three investigations are far from enough to determine the feasibility and safety of the in vivo use of GEMs. In addition to the impact on blood and tissue HM levels, a series of animal experiments on the long-term effects of GEMs on HM accumulation, metabolism, neurotoxicity, and organ damage are still required to establish a greater degree of accuracy on this matter. More broadly, some elaborate, epidemiological, and clinical studies should be extrapolated in humans to provide definitive evidence for future development. Further, the selection of suitable probiotic strains for genetic modifications, horizontal gene transfer between GEMs and other natural gut microbiota, and the dose/time-dependent effect of GEMs on the human body are still major issues in need of further investigation.

## Figures and Tables

**Table 1:** The HM-induced changes in gut microbiota composition and metabolic profiles.

Heavy Metal	The Effects on Gut Microbiota Composition	The Effects on Metabolic Profiles	Reference
As	Erysipelotrichaceae↑ Clostridiaceae↓, Catabacteriaceae↓ Cyanobacteria↓	The secretion of bile acids, amino acid, lipids, fatty acids, glucuronide, isoflavones and indole derivatives were altered	[18]
	Clostridium sulfatireducens↑ L. johnsonii↑ Butyricicoccus↑ Parasporobacterium↑ Intestinimonas↓	The fecal concentration of pro-/anti-inflammatory cytokine and chemokines was increased	[89]
	Bacteroides↑, Porphyromonadaceae↑ Lactobacillus↑ Lachnospiraceae↓ Ruminococcaceae↓	The metabolism of nitrogen and amino acid was enhanced	[90]
Cd	Clostridium_XlVb↓ Syntrophococcus↓ Cellulosilyticum↓ Prevotella↑	The amino acid and bile acid secretions were altered	[23,82,91]
	Bacteroides↑ Shewanella↑ Anaerorhabdus↑ Alistipes↑ Chryseobacterium↑ Hafnia↓, Buttiauxella↓ Arcobacter↓	The metabolism of carbohydrate, amino acid and nucleotide were promoted	[92]
Pb	Ruminococcaceae↓ Lachnospiraceae↓ Oscillibacter↓ Anaerotruncus↓ Lachnoclostridium↓	-	[93]
	Desulfovibrionaceae↑ Enterorhabdus↓ Pseudomonas↓ Desulfovibrio↓	-	[81]
	Ruminococcus↓ Coprococcus↓ Oscillospira↓ Blautia↓	The production of vitamin E and bile acids was reduced and the nitrogen and energy metabolism was altered, also induction of oxidative stress	[94]
Hg	Sutterellaceae↓ Desulfovibrionaceae↑ Helicobacteraceae↑ Rhodospirillaceae↑	Amino acid, carbohydrate, and lipid were disrupted	[80]
	Xanthomonadaceae↑ Acinetobacter↑ Nocardia↓ Aeromonas↑ Comamonadaceae families↑ Pseudomonas↑	Lipid metabolism and secretion of neurotransmission was altered	[95]

↑: Composition of the gut microbiota creased. ↓: Composition of the gut microbiota decreased.

**Table 2:** Heavy metal detoxification by probiotics and their possible mechanisms.

Probiotics	Heavy Metals	Mechanism	Reference
*Xanthomonadaceae*, *Comamonadaceae*, *Pirellula*, *Cloacibacterium*, *Deltaproteobacteria* FAC87	Hg	Convert methylated Hg to Hg^0^ that reduces its absorption	[79,89]
*Lactobacillus plantarum* (*L. plantarum*) TW1-1	Cd	Convert Cd into a less absorbable form and reduce its intra-intestinal absorption	[23]
sulfate-reducing bacteria (SRB), Fe-reducing bacteria *methanogens*, *Desulfovibrio* spp.	As, Cd, Fe	Chemical modification of HMs by methylation	[63]
*L. plantarum* CCFM8610, CCFM 8611, and *Bacillus cereus*	Hg	Increase the HM excretion accompanied by bile acid production	[23,24]
*L. plantarum* LC-705 and *Propionibacterium freudenreichii*	Pb, Cd	Decrease the intestinal PH	[90]
*Pseudomonas*, *Oxalobacter formigens (O. formigens)*	Pb, Cd, Hg, Cr, As	Form insoluble complex with HMs via siderophores and hydrogen sulfide	[92,93]
*Faecalibacterium prausnitzii (F. prausnitzii)*, *Bacteroides and Faecalibacterium*	As	Synthesize As-detoxifying enzymes	[27,94,95]
*L. plantarum* CCFM639, *Bacillus Coagulans (B. coagulans)*	As, Cd, Pb	Promote the expression of antioxidant-related genes to synthesis antioxidative enzymes	[99]
*L. plantarum* CCFM639 [100], CCFM8610 [23], *L. brevis* 23017, *Nocardia* and *Bacteroidales*	Hg	Release the HM-induced inflammatory responses by reducing the levels of proinflammatory cytokines	[23,101,102]
*Pediococcus pentosaceus* GS4, *Akkermansia muciniphila (A. muciniphila)* and *Lactobacillus rhamnosus (L. rhamnosus)* GR-1	Cd, Pb	Re-establishing the structural balance by reverse the HM-induced compositional changes in gut microbiota	[19,23,102,104]

## Data Availability

Not applicable.
